# Changes in intestinal gene expression and microbiota composition during late pregnancy are mouse strain dependent

**DOI:** 10.1038/s41598-018-28292-2

**Published:** 2018-07-03

**Authors:** Marlies Elderman, Floor Hugenholtz, Clara Belzer, Mark Boekschoten, Bart de Haan, Paul de Vos, Marijke Faas

**Affiliations:** 1 Top Institute Food and Nutrition, Wageningen, The Netherlands; 2 Division of Medical Biology, department of Pathology and Medical Biology, University of Groningen, Groningen, The Netherlands; 3 Laboratory of Microbiology, Wageningen University and Research, Wageningen, The Netherlands; 4 Division of Human Nutrition, Wageningen University and Research, Wageningen, The Netherlands; 5 Department of Obstetrics and Gynaecology, University of Groningen and University Medical Centre Groningen, Groningen, The Netherlands

## Abstract

Hormones and placental factors are thought to underlie the maternal immunological changes during pregnancy. However, as several intestinal microbiota are linked to immune modulations, we hypothesized that the intestinal microbiota are altered during pregnancy in favor of species associated with pregnancy associated immune modulations. We studied the fecal microbiota composition (MITchip) and intestinal and peripheral immune cells (microarray and flow cytometry) in pregnant and non-pregnant C57BL/6 and BALB/c mice. Pregnancy influenced intestinal microbiota diversity and composition, however in a mouse strain dependent way. Pregnant BALB/c mice had, among others, a relative higher abundance of *Lactobacillus paracasei et rel*., *Roseburia intestinalis et rel*. and *Eubacterium hallii et rel*., as compared to non-pregnant BALB/c mice, while the microbiota composition in B6 mice hardly changed during pregnancy. Additionally, intestinal immunological pathways were changed during pregnancy, however again in a mouse strain dependent way. Correlations between various bacteria and immunological genes were observed. Our data do support a role for the microbiome in changing immune responses in pregnancy. However, other factors are also involved, such as for instance changes in SCFA or changes in sensitivity to bacteria, since although immunological changes are observed in B6 mice, hardly any changes in microbiota were found in this strain. Follow up studies are needed to study the exact relationship between these parameters.

## Introduction

Pregnancy is associated with immunological adaptations of the mother in order to tolerate and support the development of the semi-allogeneic fetus. Changes in both in the innate and the specific immune response are reported during pregnancy. A decrease in the T helper 1 (Th1)/T helper 2 (Th2) ratio^1,2^ and in natural killer (NK) cells, and an increase in regulatory T cell (Treg) numbers^3,4^ has been found during pregnancy. It is known that peripheral immune responses are influenced by the placenta by direct contact with fetal tissue in the placenta, due to circulation of the immune cells through the placenta, or due to contact with soluble products produced by the placenta, such as cytokines, micro-particles and exosomes^5–7^. However, recent studies showed that the intestinal microbiome changes during pregnancy, which may also underlie the immunological changes during pregnancy^8^.

Modulation of immune cells can be induced by microbiota species present in the intestinal lumen^9–12^. Segmented filamentous bacteria for example showed to be able to induce the development of intestinal T helper 17 (Th17) cells^13^, while several bacteria species from the genus *Clostridium* have been shown to promote the accumulation of intestinal Tregs^9,10^. Additionally, *Lactobacillus plantarum* has been shown to enhance the number of peripheral Tregs^12^. One of the mechanisms by which bacteria can modulate immune cells is by producing short chain fatty acids (SCFA), such as butyrate, which has been shown to induce the differentiation of Tregs in the colon^14^. This is particularly true for *Clostridium* species from the clusters IV and XIVa^9^. However, whether intestinal bacteria are also involved in changes in peripheral immune cells during pregnancy, and whether intestinal immune cells are involved in this, is unknown.

In this study we investigated a possible relationship between microbiota and the immunological adaptations at the end of pregnancy in mice. To this end, we studied the effect of pregnancy on the microbiota composition and the effect of pregnancy on both intestinal immune cells and peripheral immune cells with a microarray (colon tissue) and flow cytometry (spleen and mesenteric lymph nodes (MLN)). We combined the transcriptomics data and the microbiota data and performed a bio-mathematical analysis, in order to find bacterial species, which may potentially be related to pregnancy induced differences in intestinal immunity. Non-pregnant mice served as control. We used two different mouse strains (C57BL/6 and BALB/c) that were shown to have different microbiota profiles and intestinal immunological responses^15,16^, to study the consequences of a different pre-pregnancy starting point in the intestinal microbiota-host interactions.

## Results

### Intestinal microbiota composition changed during pregnancy, but in a mouse strain dependent way

**Figure 1:**
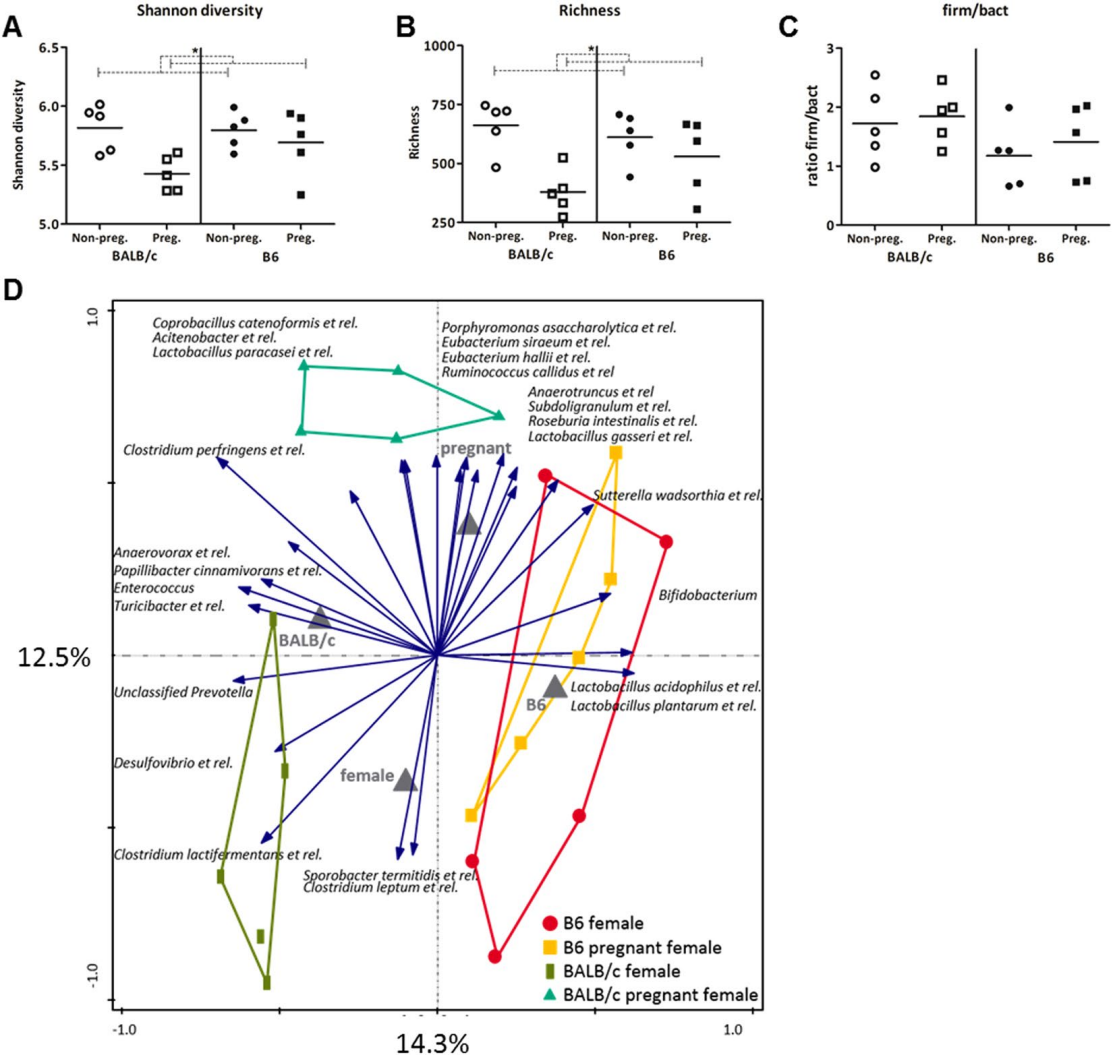
Effect of pregnancy on fecal microbiota characteristics. Shannon diversity (**A**), richness (**B**) and the firmicutes/bacteroidetes ratio (**C**) in the fecal microbiota of pregnant and non-pregnant BALB/c and B6 (n = 5). Results are shown as dot plots + means and were tested for overall strain and pregnancy effects using a Two-way ANOVA, followed by a Bonferroni post-hoc test to test for strain specific pregnancy effects when interaction was found. Significant pregnancy effects are indicated with dashed lines (*p* < 0.05). RDA plot (**D**) showing the variation explained by the components genotype and pregnancy (n = 5). The total variation that can be explained by the variables genotype (14.3%) and pregnancy (12.5%) is 26.8%. Both variables are significant in explaining the variation (Monte Carlo permutation, *p* < 0.05).

We determined the microbiota composition of non-pregnant and pregnant mice in both strains using the phylogenetic microarray, the mouse intestinal tract Chip (MITChip). We determined the richness (number of unique species) and Shannon diversity (calculation between richness and evenness) of the microbiota composition. The data was analyzed with a Two-way ANOVA (TWA), to assess if there was an interaction between pregnancy and strain on the microbiota composition in the colon. A Bonferroni post-test was performed when interaction was found, to determine whether the effect of pregnancy was the same in both mouse strains. Overall, pregnancy decreased both microbiota diversity and richness (TWA, p < 0.05) (Fig. 1A,B), but the ratio of Firmicutes/Bacteroidetes was not influenced by pregnancy (Fig. 1C).

Redundancy analysis showed that the total variation in microbiota composition explained by the variables genotype and pregnancy is 26.8%. Pregnancy explained 12.5% of the variance in microbiota composition (Fig. 1D). However, pregnancy-induced differences in microbiota at species like level were mainly observed in BALB/c mice. In B6 mice, pregnancy did not affect the microbiota at species like level. Pregnant BALB/c mice had a significantly relative higher abundance of, among others, *Allobaculum et rel*., *Lactobacillus salivarius et rel., Lactobacillus plantarum et rel., Unclassified Clostridiales XVI, Clostridium perfringens et rel., Lactobacillus paracasei et rel*., and *Roseburia intestinalis et rel*., as compared to non-pregnant BALB/c mice (Table 1).

**Table 1:** Relative abundance of bacteria groups in pregnant (P) and non-pregnant (NP) BALB/c and B6 mice (n = 5).

Bacteria group	BALB/c NP	BALB/c P	p-value	B6 NP	B6 P	p-value
*Acholeplasma et rel*.	0.0041%	0.0111%	**0.015**	0.0084%	0.0071%	1.0
*Acitenobacter et rel*.	0.0137%	0.0319%	**0.015**	0.0168%	0.0179%	1.0
*Aerococcus urinaeequi et rel*.	0.0074%	0.0176%	**0.015**	0.0098%	0.0095%	1.0
*Akkermansia muciniphila*	0.6574%	0.1152%	0.879	0.2258%	0.2113%	1.0
*Alistipes et rel*.	0.9156%	0.5594%	0.599	0.5414%	0.6029%	1.0
*Allobaculum et rel*.	0.2716%	2.4618%	**0.015**	4.0636%	2.5377%	1.0
*Anaerotruncus et rel*	2.4220%	3.8592%	**0.015**	2.3363%	5.1954%	1.0
*Anaerovorax et rel*.	0.0644%	0.0887%	0.359	0.0513%	0.0489%	1.0
*Atopobium*	0.0046%	0.0117%	**0.015**	0.0096%	0.0068%	1.0
*Bacteroides distasonis et rel*.	0.1100%	0.0565%	0.738	0.1126%	0.1959%	1.0
*Bacteroides fragilis et rel*.	0.3419%	0.1694%	0.268	0.2096%	0.5525%	1.0
*Bacteroides plebeius et rel*.	0.0077%	0.0125%	**0.047**	0.0092%	0.0095%	1.0
*Bacteroides splanchnicus et rel*.	0.4203%	0.1146%	0.078	0.5594%	0.2448%	1.0
*Bacteroides vulgatus et rel*.	0.2397%	0.0670%	0.128	0.0631%	0.0801%	1.0
*Bifidobacterium*	0.0828%	2.5962%	0.268	1.8595%	1.0711%	1.0
*Bilophila et rel*.	0.0179%	0.0154%	0.482	0.0155%	0.0131%	1.0
*Bryantella et rel*.	7.7323%	10.3212%	0.268	6.0111%	8.3875%	1.0
*Butyrivibrio crossotus et rel*.	0.4557%	0.2411%	0.197	0.5339%	0.3743%	1.0
*Butyrivibrio crossutus et rel*.	0.1210%	0.2188%	**0.025**	0.1462%	0.1571%	1.0
*Catenibacterium*	0.0140%	0.0429%	**0.015**	0.0259%	0.0210%	1.0
*Clostridium difficile et rel*.	0.9357%	2.4649%	0.078	1.1169%	0.6657%	1.0
*Clostridium herbivorans et rel*.	0.0083%	0.0202%	**0.015**	0.0132%	0.0132%	1.0
*Clostridium lactifermentans et rel*.	0.3127%	0.0903%	**0.015**	0.1212%	0.0873%	1.0
*Clostridium leptum et rel*.	0.5591%	0.2463%	**0.015**	0.4190%	0.3921%	1.0
*Clostridium perfringens et rel*.	2.3301%	6.9829%	**0.025**	0.3328%	0.8133%	1.0
*Clostridium sphenoides et rel*.	0.3833%	0.3184%	0.359	0.4446%	0.3786%	1.0
*Clostridium symbosium et rel*.	0.5136%	0.2852%	0.268	0.7587%	1.3211%	1.0
*Collinsella*	0.0077%	0.0204%	**0.015**	0.0130%	0.0214%	1.0
*Coprobacillus catenoformis et rel*.	0.0230%	0.0527%	**0.015**	0.0273%	0.0230%	1.0
*Coprobacillus et–rel. - Clostridium ramosum et rel*.	0.1377%	0.4412%	**0.015**	0.2859%	0.2632%	1.0
*Corynebacterium et rel*.	0.0067%	0.0183%	**0.015**	0.0108%	0.0099%	1.0
*Desulfovibrio et rel*.	0.0789%	0.0377%	**0.047**	0.0454%	0.0299%	1.0
*Dialister et rel*.	0.0027%	0.0066%	**0.015**	0.0044%	0.0046%	1.0
*Dorea et rel*.	3.4135%	1.4635%	0.128	2.3603%	1.9312%	1.0
*Eggerthella et rel*.	0.2295%	0.6148%	**0.015**	0.2927%	0.2089%	1.0
*Enterococcus*	0.2846%	0.6547%	0.738	0.1058%	0.0837%	1.0
*Escherichia coli et rel*.	0.0438%	0.0871%	**0.025**	0.0604%	0.0581%	1.0
*Eubacterium cylindroides et rel*.	0.0141%	0.0345%	**0.015**	0.0232%	0.0227%	1.0
*Eubacterium hallii et rel*.	0.0026%	0.0068%	**0.015**	0.0043%	0.0042%	1.0
*Eubacterium plexicaudatum et rel*.	4.0951%	4.9162%	0.359	3.4550%	4.8289%	1.0
*Eubacterium rectale et rel*.	0.0032%	0.0077%	**0.015**	0.0051%	0.3240%	1.0
*Eubacterium siraeum et rel*.	0.0084%	0.0282%	**0.015**	0.0135%	0.0138%	1.0
*Faecalibacterium prausnitzii et rel*.	0.0029%	0.0069%	**0.015**	0.0051%	0.0153%	1.0
*Fibrobacter succinogenes et rel*.	0.0110%	0.0228%	**0.015**	0.0187%	0.0161%	1.0
*Fusobacterium*	0.0137%	0.0218%	**0.015**	0.0169%	0.0178%	1.0
*Helicobacter*	0.0222%	0.0508%	**0.025**	0.0340%	0.0339%	1.0
*Labrys methylaminiphilus et rel*.	0.0076%	0.0177%	**0.015**	0.0116%	0.0114%	1.0
*Lachnobacillus bovis et rel*.	1.9742%	1.9333%	1.000	1.6788%	1.7966%	1.0
*Lachnospira pectinoschiza et rel*.	0.9649%	0.4575%	0.197	0.3867%	0.6122%	1.0
*Lactobacillus acidophilus et rel*.	0.0418%	0.0948%	**0.015**	0.4314%	0.4513%	1.0
*Lactobacillus delbrueckii et rel*.	0.0062%	0.0150%	**0.015**	0.0176%	0.0174%	1.0
*Lactobacillus gasseri et rel*.	0.0149%	0.0378%	**0.015**	0.0315%	0.0355%	1.0
*Lactobacillus paracasei et rel*.	0.0121%	0.0289%	**0.015**	0.0153%	0.0159%	1.0
*Lactobacillus plantarum et rel*.	0.0222%	0.0669%	**0.015**	0.3977%	0.3179%	1.0
*Lactobacillus salivarius et rel*.	0.5801%	3.0092%	**0.015**	1.1330%	0.9387%	1.0
*Lactococcus et rel*.	0.0029%	0.0069%	**0.015**	0.0048%	0.0044%	1.0
*Mucispirillum schaedleri et rel*.	0.1270%	0.0333%	1.000	0.0544%	0.0276%	1.0
*Olsenella et rel*.	0.0050%	0.0094%	**0.047**	0.1924%	0.0099%	1.0
*Papillibacter cinnamivorans et rel*.	0.1624%	0.1624%	0.599	0.0784%	0.0877%	1.0
*Pasteurella*	0.0103%	0.0243%	**0.015**	0.0159%	0.0168%	1.0
*Peptococcus niger et rel*.	0.1223%	0.0914%	0.268	0.1369%	0.1464%	1.0
*Porphyromonas asaccharolytica et rel*.	0.0202%	0.0434%	**0.015**	0.0287%	0.0294%	1.0
*Prevotella ruminicola et rel*.	0.0031%	0.0068%	**0.015**	0.0049%	0.0065%	1.0
*Propionibacterium*	0.0036%	0.0107%	**0.015**	0.0067%	0.0058%	1.0
*Pseudomonas et rel*.	0.0031%	0.0069%	**0.025**	0.0046%	0.0048%	1.0
*Rikenella et rel*.	0.1266%	0.1262%	1.000	0.1630%	0.1482%	1.0
*Roseburia intestinalis et rel*.	0.0210%	0.0416%	**0.025**	0.0324%	0.0347%	1.0
*Ruminobacter amylophilus et rel*.	0.0035%	0.0073%	**0.015**	0.0054%	0.0050%	1.0
*Ruminococcus callidus et rel*	0.0197%	0.0336%	**0.015**	0.0241%	0.0288%	1.0
*Ruminococcus obeum et rel*.	0.0063%	0.0133%	**0.025**	0.0091%	0.0091%	1.0
*Solobacterium moorei et rel*.	0.0116%	0.0311%	**0.015**	0.0231%	0.0197%	1.0
*Sphingomonas et rel*.	0.0113%	0.0226%	**0.015**	0.0162%	0.0156%	1.0
*Sporobacter termitidis et rel*.	17.1016%	3.5377%	**0.015**	8.8797%	7.8934%	1.0
*Staphylococcus aureus et rel*.	0.0171%	0.0406%	**0.015**	0.0216%	0.0217%	1.0
*Streptococcus intermedius et rel*.	0.0067%	0.0134%	**0.025**	0.0101%	0.0217%	1.0
*Subdoligranulum et rel*.	0.0046%	0.0086%	**0.015**	0.0061%	0.0086%	1.0
*Sutterella wadsorthia et rel*.	0.0127%	0.0331%	**0.015**	0.0300%	0.0476%	1.0
*Turicibacter et rel*.	0.6261%	1.3636%	0.268	0.3876%	0.1168%	1.0
*Unclassified Bacteroidetes*	0.0029%	0.0067%	**0.015**	0.0046%	0.0045%	1.0
*Unclassified Clostridiales -Close to Clostridium symbiosium et rel*.	1.0095%	0.8860%	0.599	1.1611%	1.2955%	1.0
*Unclassified Clostridiales I*	0.0617%	0.1479%	**0.015**	0.1199%	0.0958%	1.0
*Unclassified Clostridiales II*	0.1700%	0.2115%	0.128	0.1490%	0.2292%	1.0
*Unclassified Clostridiales IV*	0.0140%	0.0233%	0.078	0.0149%	0.0136%	1.0
*Unclassified Clostridiales XIVa*	13.2623%	13.2654%	1.000	11.6963%	12.1115%	1.0
*Unclassified Clostridiales–XIVa - close to Anaerostipes caccae*	0.0210%	0.0424%	**0.015**	0.0305%	0.0313%	1.0
*Unclassified Clostridiales XVI*	0.0751%	0.2249%	**0.015**	0.1577%	0.1431%	1.0
*Unclassified Cyanobacteria*	0.0046%	0.0072%	**0.025**	0.0056%	0.0052%	1.0
*Unclassified Mollicutes*	0.1218%	0.4635%	**0.047**	0.2794%	0.2072%	1.0
*Unclassified Porphyromonadaceae*	34.4807%	32.6881%	0.879	44.8531%	41.1783%	1.0
*Unclassified Prevotella*	1.1225%	0.5609%	0.599	0.2821%	0.2229%	1.0
*Unclassified TM7*	0.0124%	0.0252%	**0.025**	0.0156%	0.0156%	1.0
*Uncultured Clostridiales*	0.2248%	0.1644%	0.078	0.1670%	0.1885%	1.0
*Veilonella*	0.0026%	0.0066%	**0.015**	0.0044%	0.0054%	1.0
*Vibrio et rel*.	0.0130%	0.0334%	**0.015**	0.0218%	0.0191%	1.0

Differences between pregnant and non-pregnant mice within each mouse strain were determined with a Mann–Whitney U test. P-values were adjusted for multiple comparisons with the Benjamini-Hochberg procedure. Significant differences are highlighted in bold.

### Pregnancy influenced the expression of genes related to intestinal immune pathways in a mouse strain dependent way

Next, we determined the effect of pregnancy on intestinal immunological pathways with a microarray on colonic tissue. In the colon, 1032 genes were differentially expressed between pregnant and non-pregnant BALB/c mice, and 789 genes were differentially expressed in B6 mice. The two strains shared 161 differentially expressed genes between pregnant and non-pregnant females in the colon (both up- and down-regulated).

To gain insight into the biological role of the genes which were influenced by pregnancy, we looked into the pathways in which these genes are involved using Ingenuity Pathway Analysis (IPA), with special focus on pathways related to immunology (Table 2). In both mouse strains, IPA showed that pregnancy significantly influenced the expression of genes related to both the innate and adaptive immunity. However, in the B6 strain, the effect of pregnancy was more pronounced concerning pathways related to immunology. In the colon of B6 mice, pregnancy influenced, among others, the antigen presentation pathway, T cell co-stimulation (CD28/iCOS), dendritic cell (DC) maturation and cytokine signaling (IL-4/IL-8/IL-9). In the colon of BALB/c mice, among others, the antigen presentation pathway, DC maturation and the IL-10 signaling pathway were influenced by pregnancy (Table 2).

**Table 2:** Selection of immunological pathways that are related to the genes with a different expression in pregnant and non-pregnant females in both BALB/c and B6 mice in the proximal colon (n = 5), analyzed with Ingenuity pathways analysis.

Ingenuity Canonical Pathways	*p*-value B6	*p*-value BALB/c	z-score B6	*z*-score BALB/c
Antigen Presentation Pathway	**0.000**	**0.000**	NaN	NaN
B Cell Development	**0.001**	0.076	NaN	NaN
CD28 Signaling in T Helper Cells	**0.003**	0.259	NaN	NaN
Communication between Innate and Adaptive Immune Cells	**0.039**	**0.000**	NaN	NaN
Complement System	**0.001**	**0.022**	NaN	NaN
Crosstalk between Dendritic Cells and Natural Killer Cells	**0.010**	**0.000**	NaN	NaN
Dendritic Cell Maturation	**0.000**	**0.036**	NaN	NaN
Differential Regulation of Cytokine Production in Intestinal Epithelial Cells by IL-17A and IL-17F	**0.011**	0.143	NaN	NaN
Granulocyte Adhesion and Diapedesis	**0.000**	**0.006**	NaN	NaN
iCOS-iCOSL Signaling in T Helper Cells	**0.000**	0.095	NaN	NaN
IL-10 Signaling	0.163	**0.048**	NaN	NaN
IL-4 Signaling	**0.001**	0.379	NaN	NaN
IL-8 Signaling	**0.005**		−2.530	
IL-9 Signaling	**0.005**	0.634	NaN	NaN
Interferon Signaling	1.000	**0.000**		−2.449
Leukocyte Extravasation Signaling	**0.001**	0.219	−2.121	1.134
phagosome formation	**0.027**	**0.016**	NaN	NaN
Role of Pattern Recognition Receptors in Recognition of Bacteria and Viruses	0.120	**0.012**	−1.000	−0.447
T Helper Cell Differentiation	**0.001**	**0.017**	NaN	NaN

The *p*-value is a measure of the likelihood that the association between the set of genes altered by pregnancy in this experiment and the given pathways is due to random chance. A positive z-score indicates a predicted activation, and a negative z-score indicates a predicted inactivation of the enriched pathway. NaN indicates a z-score which cannot be calculated for the Ingenuity canonical pathway. Significant differences are highlighted in bold.

### Correlation between microbiota species and gene expression profile in the colon

**Figure 2:**
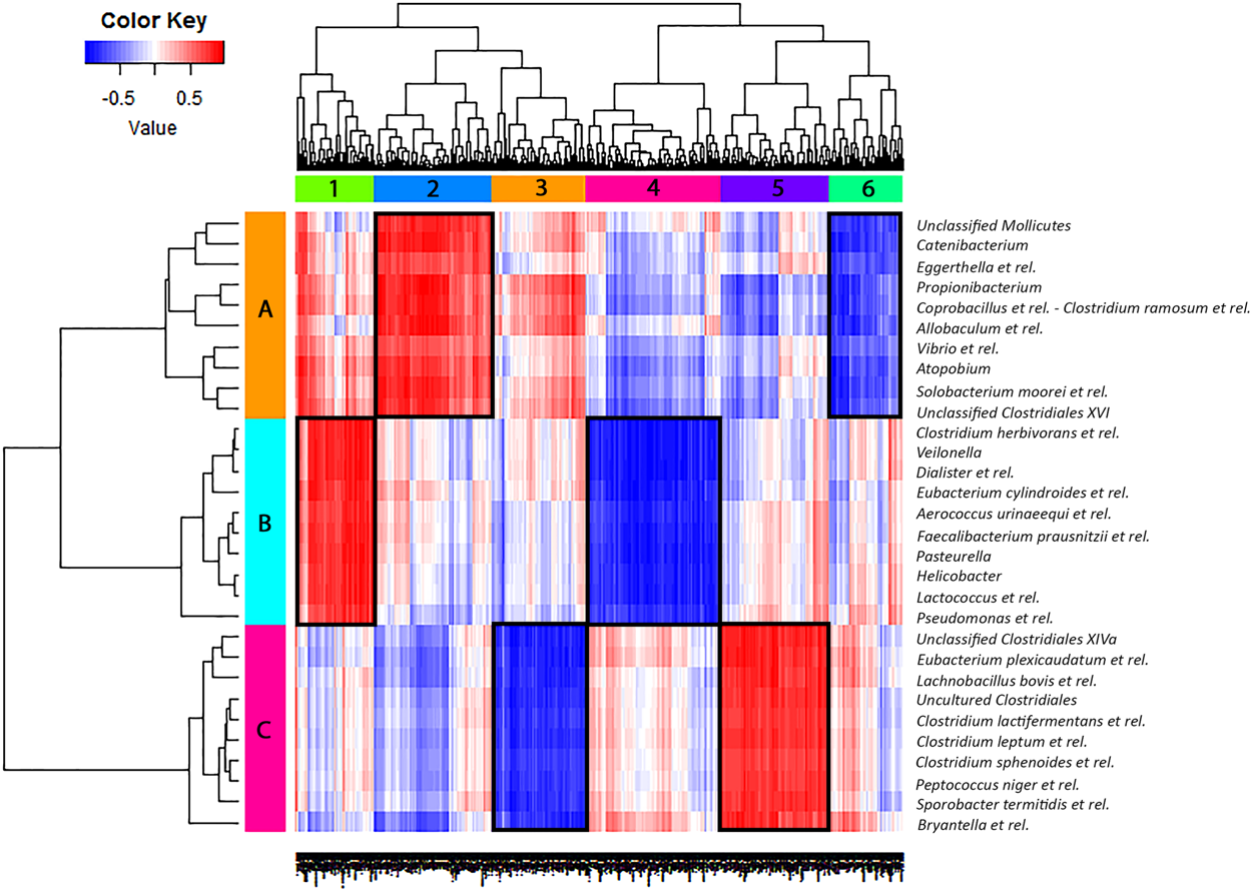
Correlation between microbiota species and gene expression in the colon. This figure shows a heat-map which correlates the MITChip data (vertical) and the microarray data (horizontal) of pregnant and non-pregnant BALB/c mice. For each mouse the datasets were integrated individually mouse (5 mice per group) and it gives the direct correlations between gene expression and microbiota composition from these samples. Cluster of genes that correlated most positively with a respective group of bacteria are indicated in deep red, while genes that correlated most negatively with a respective group of bacteria are indicated in deep blue. Clusters which are framed in black are discussed in more detail in the text. Six main gene clusters (1–6) were identified and three bacterial clusters (**A**–**C**).

To investigate the relation between microbiota species and immunological gene expression, we combined microbiota and colonic gene expression data from individual pregnant and non-pregnant BALB/c mice, to evaluate direct correlations between gene expression and microbiota composition in these samples (Fig. 2). We only did this for the BALB/c strain as no microbiota species were significantly changed during pregnancy in the B6 strain. We integrated the datasets using a PLS-based canonical correlation approach. In total 600 genes and 30 bacterial groups were retained for the first three components, and clustering of the correlation coefficients showed six main clusters of host genes that correlated negatively (blue) or positively (red) with three main clusters of bacteria.

Strongest correlations were found between bacterial cluster A with gene expression cluster two and six, bacterial cluster B with gene expression cluster one and four and bacterial cluster C with gene expression cluster three and five. All bacteria in cluster A (such as *Allobaculum et rel*. and *Unclassified Mollicutes*) had a higher relative abundances in pregnant BALB/c mice as compared to non-pregnant BALB/c mice. Gene cluster two showed a positive correlation, while gene cluster six showed a negative correlation with bacteria in cluster A. Some genes in cluster two and six were involved in immunological functions, particularly in T cell development (Supplementary Table S1). Also all bacteria in cluster B (such as *Eubacterium cylindroides et rel*. and *Faecalibacterium prausnitzii et rel*.) had a higher relative abundances in pregnant BALB/c mice as compared to non-pregnant BALB/c mice. Gene cluster one showed a positive correlation, while gene cluster four showed a negative correlation with bacteria in cluster B. However, almost no genes from cluster one and four were involved in immunological functions (Supplementary Table S2). Interestingly, cluster C contained most of the bacteria which had a significantly lower abundance (*Clostridium lactifermentans et rel., Clostridium leptum et rel*. and *Sporobacter termitidis et rel*.) in pregnant BALB/c mice as compared to the non-pregnant BALB/c mice. Gene cluster three showed a negative correlation, while gene cluster five showed a positive correlation with bacteria in cluster C. Many genes from cluster three and five were involved in immunological functions, especially in lymphocyte and T cell development (Supplementary Table S3).

### Pregnancy influenced T cells in the MLN and spleen in a mouse strain dependent way

As we found effects of pregnancy on the microbiota composition and intestinal immunological gene expression using a microarray, we subsequently studied immune cell subsets in the MLN and the spleens with flow cytometry. We used the MLN as they are the draining lymph nodes for the intestines. The spleen was used as a reference for systemic immunity. We focused on several T cell subsets, as many genes which were related to pregnancy induced microbiota changes were related to T cells. Again we analyzed the data with a TWA, to understand if there was an interaction between pregnancy and mouse strain on the immune populations. When an interaction was present we tested with a Bonferroni post-test whether the effect of pregnancy was the same in both mouse strains.

**Figure 3:**
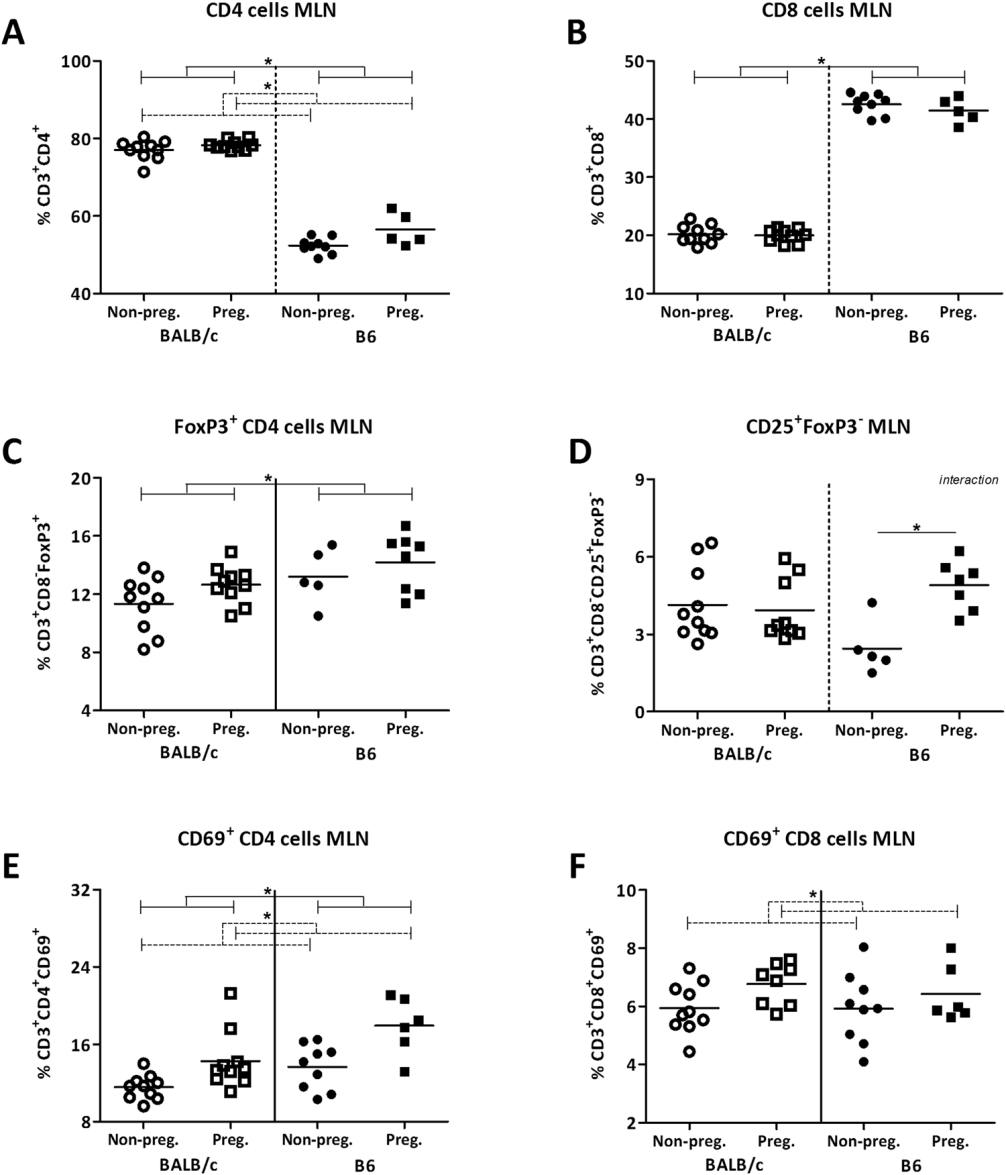
Effect of pregnancy on T cell populations in the mesenteric lymph nodes. Frequency of T helper cells (CD4^+^) (**A**), cytotoxic T cells (CD8^+^) (**B**), FoxP3^+^ Tregs (**C**), CD25^+^ FoxP3^−^ effector CD4^+^ cells (**D**), CD69^+^ CD4^+^ cells (**E**), and CD69^+^ CD8^+^ cells (**F**) in the mesenteric lymph nodes (MLN) of pregnant and non-pregnant BALB/c and B6 mice. T cytotoxic and T helper cells are expressed as the frequency of CD8^+^ and CD8^−^ cells within the CD3^+^ population respectively. Due to technical problems, several MLNs could not be used for immune cell analysis, which reduced the number of mice in these groups. Results are shown as dot plots + means and were tested for overall strain and pregnancy effects using a Two-way ANOVA, followed by a Bonferroni post-hoc test to test for strain specific pregnancy effects when interaction was found. Significant strain effects are indicated with solid lines and significant pregnancy effects are indicated with dashed lines (*p* < 0.05).

In the MLN, overall the percentage of CD4^+^ cells was higher in pregnant mice (TWA, p < 0.05) (Fig. 3A). The percentage of CD8^+^ cells in the MLN was not affected by pregnancy (Fig. 3B). Pregnant mice also tended to have a higher percentage of FoxP3^+^CD4^+^ cells in the MLN than non-pregnant mice (TWA, p = 0.0776) (Fig. 3C). Pregnancy changed the percentage of CD25^+^FoxP3^−^ CD4^+^ cells in the MLN, although in a mouse strain dependent way (interaction between pregnancy and strain TWA, p < 0.05). In pregnant B6 mice, but not in pregnant BALB/c mice, the percentage of CD25^+^FoxP3^−^ CD4^+^ cells was higher than in non-pregnant mice (Bonferroni, p < 0.05) (Fig. 3D). Additionally, the percentage of both CD4^+^ and CD8^+^ cells expressing CD69 in the MLN was higher in pregnant than non-pregnant mice (TWA, p < 0.05) (Fig. 3E,F).

**Figure 4:**
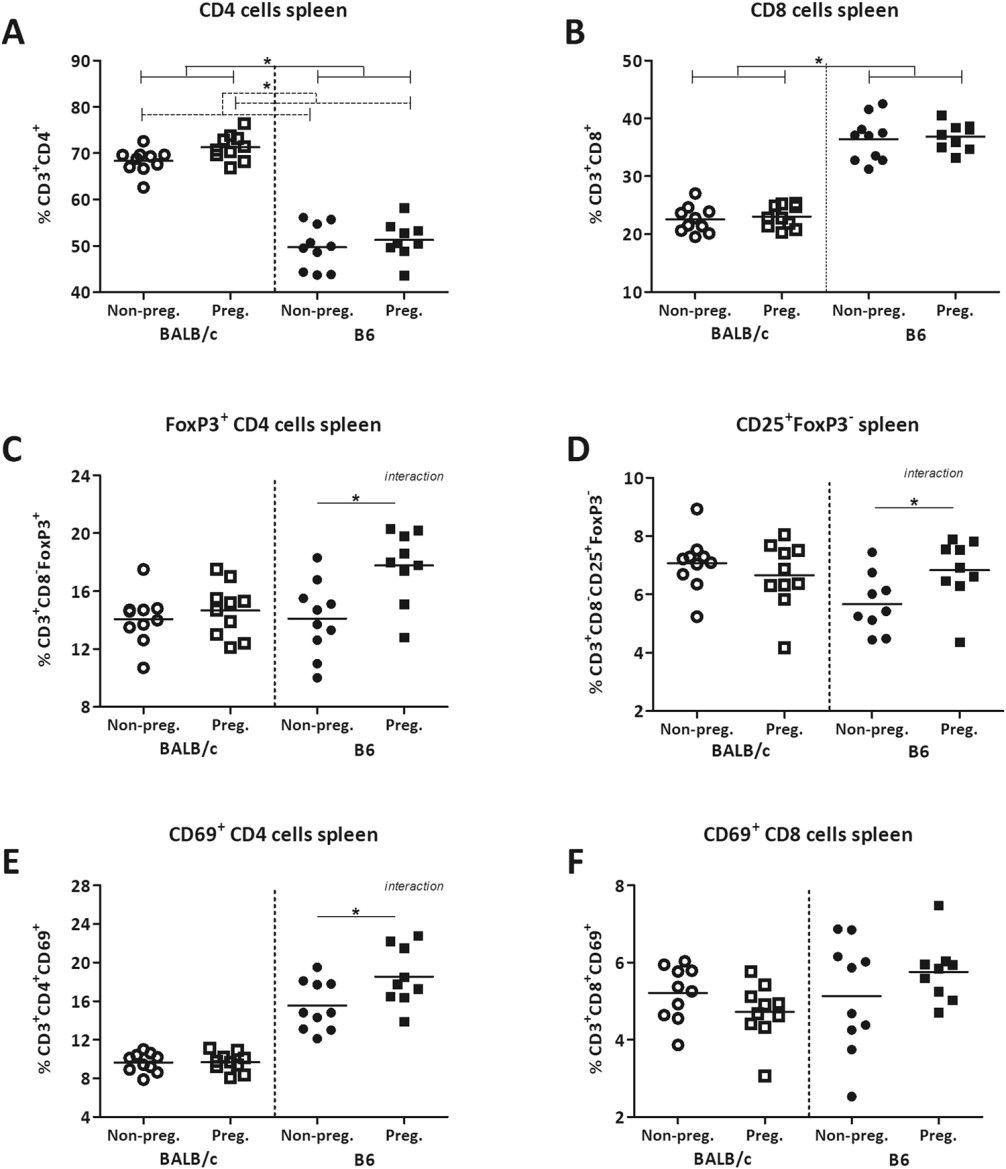
Effect of pregnancy on T cell populations in the spleen. Frequency of T helper cells (CD4^+^) (**A**), cytotoxic T cells (CD8^+^) (**B**), FoxP3^+^ Tregs (**C**), CD25^+^FoxP3^−^ effector CD4^+^ cells (**D**), CD69^+^ CD4^+^ cells (**E**), and CD69^+^ CD8^+^ cells (**F**) in the spleens of pregnant and non-pregnant BALB/c and B6 mice. T cytotoxic and T helper cells are expressed as the frequency of CD8^+^ and CD4^+^ cells within the CD3^+^ population respectively. Results are shown as dot plots + means and were tested for overall strain and pregnancy effects using a Two-way ANOVA, followed by a Bonferroni post-hoc test to test for strain specific pregnancy effects when interaction was found. Significant strain effects are indicated with solid lines and significant pregnancy effects are indicated with dashed lines (*p* < 0.05).

In the spleen, pregnant mice had an overall higher percentage of CD4^+^ cells than non-pregnant mice (TWA, p < 0.05), while pregnancy had no effect of on the percentage of CD8^+^ cells (Fig. 4A,B). Pregnancy also influenced the percentage of FoxP3^+^CD4^+^ cells in the spleen (Fig. 4C), however, in a mouse strain dependent way (interaction between pregnancy and strain in TWA, p < 0.05). The percentage of FoxP3^+^CD4^+^ cells was not changed in pregnant BALB/c mice as compared to non-pregnant BALB/c mice, whereas pregnant B6 mice had a significantly higher percentage of FoxP3^+^CD4^+^ cells than non-pregnant B6 mice (Bonferroni, p < 0.05). Pregnancy also affected the percentage of CD25^+^FoxP3^−^ CD4^+^ cells in the spleen (Fig. 4D), again in a mouse strain depended way (interaction between pregnancy and strain in TWA, p < 0.05). Only pregnant B6 mice, but not pregnant BALB/c mice, had a significantly higher percentage of CD25^+^FoxP3^−^ CD4^+^ cells than non-pregnant B6 mice (Bonferroni, p < 0.05). The percentage of CD4^+^ cells expressing the early activation marker CD69 was affected by pregnancy in the spleen, although also in a mouse strain dependent way (interaction between pregnancy and strain, TWA, p < 0.05) (Fig. 4E): the percentage of CD4^+^ cells expressing CD69 was not affected in pregnant BALB/c mice, while it was significantly higher in pregnant B6 mice than non-pregnant mice (Bonferroni, p < 0.05). Pregnancy did not alter the percentage of CD8^+^ expressing CD69 (Fig. 4F) in the spleen.

### Pregnancy increased CD103 DCs in the mesenteric lymph nodes

**Figure 5:**
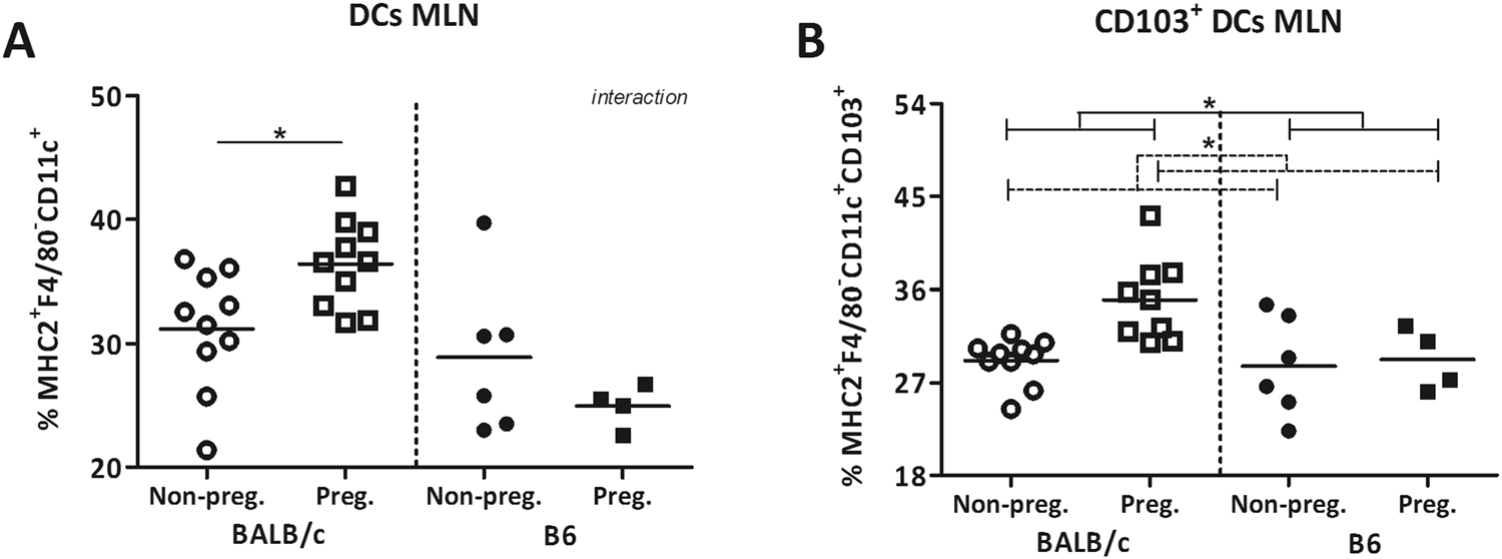
Effect of pregnancy on CD103^+^ dendritic cells in the mesenteric lymph nodes. Percentage of dendritic cells (DCs) (**A**) and their expression of CD103 (**B**) in the mesenteric lymph nodes (MLN) of pregnant and non-pregnant BALB/c and B6 mice. DCs are expressed as the percentage of CD11c^+^ cells within the F4/80^−^MHC2^+^ population. Due to technical problems, several MLNs could not be used for immune cell analysis, which reduced the number of mice in these groups. Results are shown as dot plots + means and were tested for overall strain and pregnancy effects using a Two-way ANOVA, followed by a Bonferroni post-hoc test to test for strain specific pregnancy effects when interaction was found. Significant strain effects are indicated with solid lines and significant pregnancy effects are indicated with dashed lines (*p* < 0.05).

There was an interaction between pregnancy and strain for the percentage of DCs in MLN (TWA, p < 0.05) (Fig. 5A). The percentage of DCs expressing CD103 was higher in pregnant mice than non-pregnant mice (TWA, p < 0.05) (Fig. 5B).

## Discussion

Pregnancy is associated with peripheral immune changes^3,4^. We hypothesized that this may be related to changes in the gut microbiome. We found that pregnancy influenced the intestinal microbiota composition as well as the expression of genes related to immunological pathways in the colon, but in a mouse strain dependent way.

We demonstrate that pregnancy impacts the intestinal microbiota composition in mice, however, in a mouse strain depended way. We showed that at the end of pregnancy, pregnant mice had a lower microbiota diversity and richness than non-pregnant mice, but this decrease was more pronounced in BALB/c mice. Our data appear to be in line with a human study, which demonstrated a decreased diversity in the third trimester as compared to the first trimester of pregnancy in humans^8^. In general, it is assumed that a microbiome with a low diversity and richness is not beneficial for the host health, as disorders like obesity are general associated with a low microbial diversity and richness^17,18^. It remains therefore to be investigated why diversity and richness are decreased in pregnancy. However, it might be a functional change as Koren *et al*. showed that transferring microbiota from third trimester pregnant women to germ-free mice, induced adiposity and insulin insensitivity in these mice^8^. Therefore, it may be suggested that during pregnancy, one of the functions of the changes in microbiome is enhancing energy absorption in the mother.

When looking at bacterial species like level, we also found changes during pregnancy, however only in the BALB/c strain. During pregnancy, BALB/c mice had a higher abundance of, among others, *Eubacterium hallii et rel., Lactobacillus paracasei et rel., Lactobacillus plantarum et rel*., and *Roseburia intestinalis et rel*. These species have been shown to modulate immune responses, and are especially associated with immune tolerance and anti-inflammatory properties^12,19–21^. *Lactobacillus paracasei et rel*. has been shown to increase the amount of both CD103 expressing DCs, i.e. DCs inducing immune tolerance, and Tregs in the MLN of mice^21^. This is in accordance with the higher percentage of CD103^+^ DCs we found in the MLNs of pregnant BALB/c mice. *Eubacterium hallii*, as well as *Roseburia intestinalis* are well known butyrate producers^19,20^, which can induce the differentiation of Tregs in the lamina propria of the colon^14^. Species of the *Lactobacillus* genus are well known for their beneficial effects on health, including the fact that they can upregulate Tregs and have anti-inflammatory properties^12^.

Interestingly, in contrast to the BALB/c strain, pregnancy induced changes in microbiota composition were less apparent in the B6 strain. This may be in contradiction with a study of Gohir *et al*. (2015), who found a shift in microbiota composition in B6 mice during pregnancy, however they took fecal samples at earlier time-points (day 5, day 10 and day 15 of gestation), which may explain the different findings^22^. A potential explanation for the lack of changes in the B6 microbiome during pregnancy maybe the fact that non-pregnant B6 females, in contrast to non-pregnant BALB/c, already had a high abundance of bacteria from the *Lactobacillus* genus. Bacteria from this genus increase in pregnant BALB/c mice. Therefore, there may be no need for changes in bacterial composition during pregnancy in the B6 mice. Despite the lack of changes in the B6 microbiome, peripheral Tregs were increased in these mice during pregnancy. This may indicate that changes in Tregs in B6 mice are induced by other factors than microbiota or that pregnant B6 mice are more sensitive to the effects of microbiota, such as bacteria from the *Lactobacillus* genus. Additionally, a change in bacterial metabolic products during pregnancy, such as SCFA, may also underlie the change in Tregs in B6. These hypotheses need to be tested in future studies, and currently we investigate the immune modulatory effects of the specific pregnancy enriched bacteria in germ free mice.

We also evaluated the effect of pregnancy on the expression of genes involved in immune pathways in the colon. By using a microarray and Ingenuity Pathway Analysis, we demonstrated that pregnancy altered the expression of many genes involved in immune pathways in the colon. In both mouse strains we found immunological pathways which were differentially regulated by pregnancy. These include, among others, the antigen presenting pathway, DC maturation, T helper cell differentiation and IL-10 signaling. The effect of pregnancy on these pathways, however, was dependent on the mouse strain. Some pathways were modulated by pregnancy in both strains, such as DC maturation and T helper cell differentiation. However, other pathways were only modulated by pregnancy in B6 mice, such as the pathways related to CD28/ICOS, or were only modulated by pregnancy in BALB/c mice, such as interferon signaling. Despite the lack of changes in the B6 microbiome, the expression of immunological genes in the colon was also changed during pregnancy. Again, this may indicate that changes in the immune system in B6 mice are induced by other factors than microbiota or that immunological genes in pregnant B6 mice are more sensitive to the modulating effects of microbiota.

As the present study was an observational study, more experimental data are needed in order to state that the microbiome is driving immune changes during pregnancy. However, our data do support a role for the microbiome in immunological changes during pregnancy, as our data show that the presence of several bacterial species which were up- or down-regulated during pregnancy in BALB/c mice were correlated to clusters of genes in the colon (Fig. 2), of which many are related to immunological functions, such as T cell development. With flow cytometry we aimed to confirm the immunological changes found in the intestine, and indeed we found changes in T cell populations in both the MLN and spleen during pregnancy, however again in a mouse strain dependent way. We demonstrated that pregnancy tended (p = 0.0776) to increase Treg cell numbers in the MLNs, while the percentage of CD103^+^ DCs in the MLN was only increased in pregnant BALB/c mice. DCs expressing CD103 in the MLN are able to induce the generation of FoxP3^+^ Treg cells^23^. Interestingly, in our study there does not seem to be a relation between CD103^+^ DCs and FoxP3^+^ Tregs, as in the BALB/c mice we found a higher percentage of intestinal CD103^+^ DC in pregnant mice, while the percentage of peripheral Tregs was not increased; in B6 mice we found an increased percentage of peripheral Tregs in pregnant mice, while the percentage of intestinal CD103^+^ DCs was not increased. The reason for this discrepancy is unknown, however, it may be due to differences in expression of co-factors such as TGF-ß or retinoic acid. These cofactors are necessary for CD103^+^ DCs to drive Treg generation^24^. Another explanation may be that the induced Tregs are not targeted to the spleen but recruited to the uterus draining lymph nodes^25^.

In summary, our data showed changes in microbiota composition and expression of genes related to immunological pathways in the colon, during late pregnancy. Although our data do support a potential role for the microbiome in changing immune responses in pregnancy, other factors are also involved, such as for instance changes in SCFA or changes in sensitivity to bacteria, since although immunological changes are observed in B6 mice, hardly any changes in microbiota were found in this strain. Further studies are needed to evaluate the exact relationship between these parameters. Measurements, such the sensitivity of the immune system for bacteria, immune cells in the decidua and earlier time-points in pregnancy should be included in follow up studies to further investigate the relation between the microbiome and pregnancy induced immune adaptations.

## Materials and Methods

### Mice

All animal experiments were performed after receiving approval of the Animal Care Committee of the Groningen University (DEC#6349 A) and we confirm that all methods were performed in accordance with their guidelines and regulations. Female wild-type B6 and BALB/c mice (n = 20 per strain) were purchased from Harlan (Harlan, Horst, the Netherlands) at an age of eight weeks. All mice were co-housed (four cages, five mice per cage, pregnant and non-pregnant mice were separately housed) in isolated ventilated cages to limit environmental influences. The animals had ad libitum access to a D12450B diet (Research Diets Services, Wijk bij Duurstede, the Netherlands) and water. This diet was given immediately after the mice arrived in the animal facility, this was at least three weeks before sacrifice. Between an age of 12 weeks and 32 weeks (mean age of the mouse per groups is given in Tables 3 and 4) all mice were sacrificed by cervical dislocation under anesthesia. All non-pregnant female mice (n = 10 from each strain) were sacrificed during the diestrus phase of their ovarian cycle to ensure low stable levels progesterone and estrogens. For the pregnant mice, smears were taken daily and, when in the proestrus phase of their ovarian cycle, they were placed (overnight) in a cage with a male wild-type mice from the same strain and placed back in their original cage the next morning. The day of the vaginal plug was day 0 of pregnancy. Pregnant mice were sacrificed at day 18 of gestation. At sacrifice, spleens and MLN’s were removed for immune cell analysis. Approximately 1 cm of proximal colon was frozen in liquid nitrogen and stored at −80 °C prior to RNA isolation and transcriptomics. Feces from the last part of the colon were collected for microbiota profiling. Table 3 provides an overview of the characteristics of all mice and Table 4 provides an overview of the characteristics of the mice which were, per strain, randomly selected for microbiota and gene array analysis (n = 5).

**Table 3:** Overview of mice characteristics.

	non-pregnant B6	pregnant B6	non-pregnant BALB/c	pregnant BALB/c
Number of mice	10	9*	10	10
Weight before pregnancy (gr)		21.4 (2.0)		22.0 (1.7)
Weight at sacrifice (gr)	22.4 (1.1)^a^	34.5 (2.4)^b^	22.2 (1.4)^a^	34.4 (4.9)^b^
Age at sacrifice (weeks)	18.4 (2.2)^abc^	22.8 (5.5)^b^	16.5 (4.1)^c^	18.6 (3.9)^abc^
Average number of pups		6.6 (2.3)		6.4 (3.4)

The letters a, b and c indicate which groups differ significantly (One-way Anova followed by Dunn’s multiple comparison test, *p* < 0.05) from each other. *One pregnant B6 mouse died preliminary.

**Table 4:** Overview of mice characteristics selected for microbiota and gene array analysis.

	non-pregnant B6	pregnant B6	non-pregnant BALB/c	pregnant BALB/c
Number of mice	5	5	5	5
Weight before pregnancy (gr)		19.9 (1.6)^a^		22.6 (1.4)^b^
Weight at sacrifice (gr)	22.1 (1.0)^a^	33.6 (1.4)^b^	22.1 (1.0)^a^	35.5 (5.3)^b^
Age at sacrifice (weeks)	18.2 (0.4)	19.9 (4.2)	18.5 (3.5)	15.6 (0.6)
Average number of pups		8.0 (1.5)		7.1 (4.4)

The letters *a* and b indicate which groups differ significantly (One-way Anova followed by Dunn’s multiple comparison test, *p* < 0.05) from each other.

### Bacterial DNA extraction and microbiota profiling

Luminal contents from the last part of the distal colon (n = 5 per group) were analyzed by a Mouse Intestinal Tract Chip (MITChip), as previously described^26^. The Redundancy analysis (RDA) was performed in Canoco 5.0, and visualized in triplots or a PRC plot^27^.

### Intestinal microarray analysis

RNA was purified from the proximal colon of mice (n = 5 per group) and RNA expression profiling was performed with an Affymetrix Mouse Gene 1.1 ST array plate (Affymetrix, Santa Clara, CA, USA) as previously described^26^. Only probe sets with a fold-change of at least 1.2 (up/down) and a p-value < 0.05 were considered to be significantly different.

To gain insight into the biological role of the differently expressed genes during pregnancy, we investigated the pathways in which these genes are involved using Ingenuity Pathway Analysis (IPA) (Ingenuity System). Our IPA analyses included comparison of differentially regulated genes in the colon of pregnant and non-pregnant B6 and BALB/c mice.

### Multivariate integration and correlation analysis

To gain insight in the relationship between the genes differently expressed during pregnancy in the colon and the microbiota composition in BALB/c mice, the microarray and MITChip datasets were combined, using the linear multivariate method partial least squares (PLS)^28^, as described previously^29^. This integration of datasets per individual mouse gives a direct correlation between gene expression and microbiota composition in these samples (n = 5 per group). Before analysis, the sets of data were log2 transformed, and the canonical correlation framework of PLS was used^30^. The correlation matrices were visualized in clustered image maps^31^. Analyses were performed in R using the library mixOmics^32^.

### Isolation of splenic and mesenteric lymph node cells

Single cell suspensions of spleens and MLNs were made as previously described^26^.

### Cell staining and flow cytometry

Spleen and MLN cells were stained for T cell populations. T cells were determined using CD3, T cytotoxic cells with CD8 and T helper cells with CD4. CD69 expression was determined within both the CD8^+^ and CD4^+^ cell subsets. In another panel we determined the expression of FoxP3 and CD25, using CD3 and CD8. Within the MLN, also the percentage of DCs, and DCs expressing CD103 were determined using F4/80, MHC2, CD11c and CD103. Antibody specifications are described in Supplementary Table S4.

The antibodies were diluted in a total volume of 25 µl with FACS buffer (PBS + 10% FCS (v/v)). Approximately 1 × 10^6^ spleen or MLN cells were incubated for 20 minutes in FACS buffer (10% FCS (*v/v*)) with 20% (*v/v*) normal rat serum (Jackson, Newmarket, UK) and 2% (*v/v*) Fc block (CD16/32) (Biolegend, Uithoorn, the Netherlands). Next, they were incubated with an extracellular antibody mix for 30 minutes. Then, samples were washed twice with a permeabilization buffer (eBioscience, Vienna, Austria) after which they were incubated with an intracellular blocking medium (20% (*v/v*) rat serum in permeabilization buffer (eBioscience, Vienna, Austria) for 20 minutes. Subsequently, these cells were incubated with an intracellular antibody mix for 30 minutes and fixed in FACS lysing BD Biosciences, Breda, The Netherlands) solution for 30 minutes. In between all incubation steps washing was performed all incubation steps and the whole procedure was performed on ice and in the dark. Isotype control antibodies were used at the same dilution and purchased from the same company as the extracellular and intracellular antibodies.

Cell samples were analyzed using the LSR-II Flow Cytometer system (BD Biosciences, Breda, the Netherlands), using FACS Diva software. Analysis was performed using FlowJo version 10 software (FlowJo, LLC, Oregon, USA). The gating strategy for T lymphocytes is shown in Supplementary Figure S1. To gate T cells, first lymphocytes were gated based on size in the forward side scatter plot. Next, T cells were determined by selecting CD3^+^ cells. CD4^+^ and CD8^+^ cells were selected within the CD3^+^ cells. Next, within both the CD4^+^ and CD8^+^ population, the percentage of cells expressing CD69 was measured and within the CD4^+^ cells also the percentage of cells expressing FoxP3 and CD25 was assessed (Supplementary Figure S2). To gate DCs, first living cells were selected based on size in the forward side scatter plot. Within the MHC2^+^ and F4/80^−^, DCs were gated as CD11c^+^. In this population the expression of CD103 was determined (Supplementary Figure S3). All the isotype controls were set at 1% and these gates were copied to the samples with the antibody mix.

### Statistics

For Shannon diversity, microbiota richness, the firmicutes/bacteroidetes ratio and flow cytometry, data are expressed as dot plots + means. The Kolmogorov-Smirnov test was used to determine normal distribution of the data. When the data were not normally distributed a log transformation was performed before analysis. The overall effect of pregnancy and strain was determined with a Two-way ANOVA (TWA), followed by a Bonferroni post-hoc test when an interaction between pregnancy and strain was found. For analyzing significant effects of pregnancy on the abundance of bacterial groups within each strain, a Mann–Whitney U test was used. P-values of < 0.05 were considered statistically significant and p-values between 0.05 and 0.1 were defined as a trend. P-values were adjusted for multiple comparisons with the Benjamini-Hochberg procedure.

### Availability of data and materials

The gene array datasets generated and analyzed during the current study will be available in the gene expression omnibus from NCBI under ID number xxxx.

## Electronic supplementary material

Supplementary information
