# Supplementary material for: Changes in intestinal gene expression and microbiota composition during late pregnancy are mouse strain dependent
Source: Sci Rep. 2018 Jul 3;8:10001. doi: 10.1038/s41598-018-28292-2 (PMC6030191; doi:10.1038/s41598-018-28292-2)
Supplement: Supplementary file 1 — Supplementary information [file 41598_2018_28292_MOESM1_ESM.docx]

**Supplementary information**

**Title: Changes in intestinal gene expression and microbiota composition during late pregnancy are mouse strain dependent.**

Marlies Elderman^1,2^*, Floor Hugenholtz^1,3^, Clara Belzer^1,3^, Mark Boekschoten^1,4^, Bart de Haan^2^, Paul de Vos^1,2^, Marijke Faas^2,5^

^1^ Top Institute Food and Nutrition, Wageningen, the Netherlands

^2^ Division of Medical Biology, department of Pathology and Medical Biology, University of Groningen, Groningen, The Netherlands

^3^ Laboratory of Microbiology, Wageningen University and Research, Wageningen, the Netherlands

^4^ Division of Human Nutrition, Wageningen University and Research, Wageningen, the Netherlands

^5^ Department of Obstetrics and Gynaecology, University of Groningen and University Medical Centre Groningen, Groningen, the Netherland

* m.e.elderman@umcg.nl

**Supplementary figures**


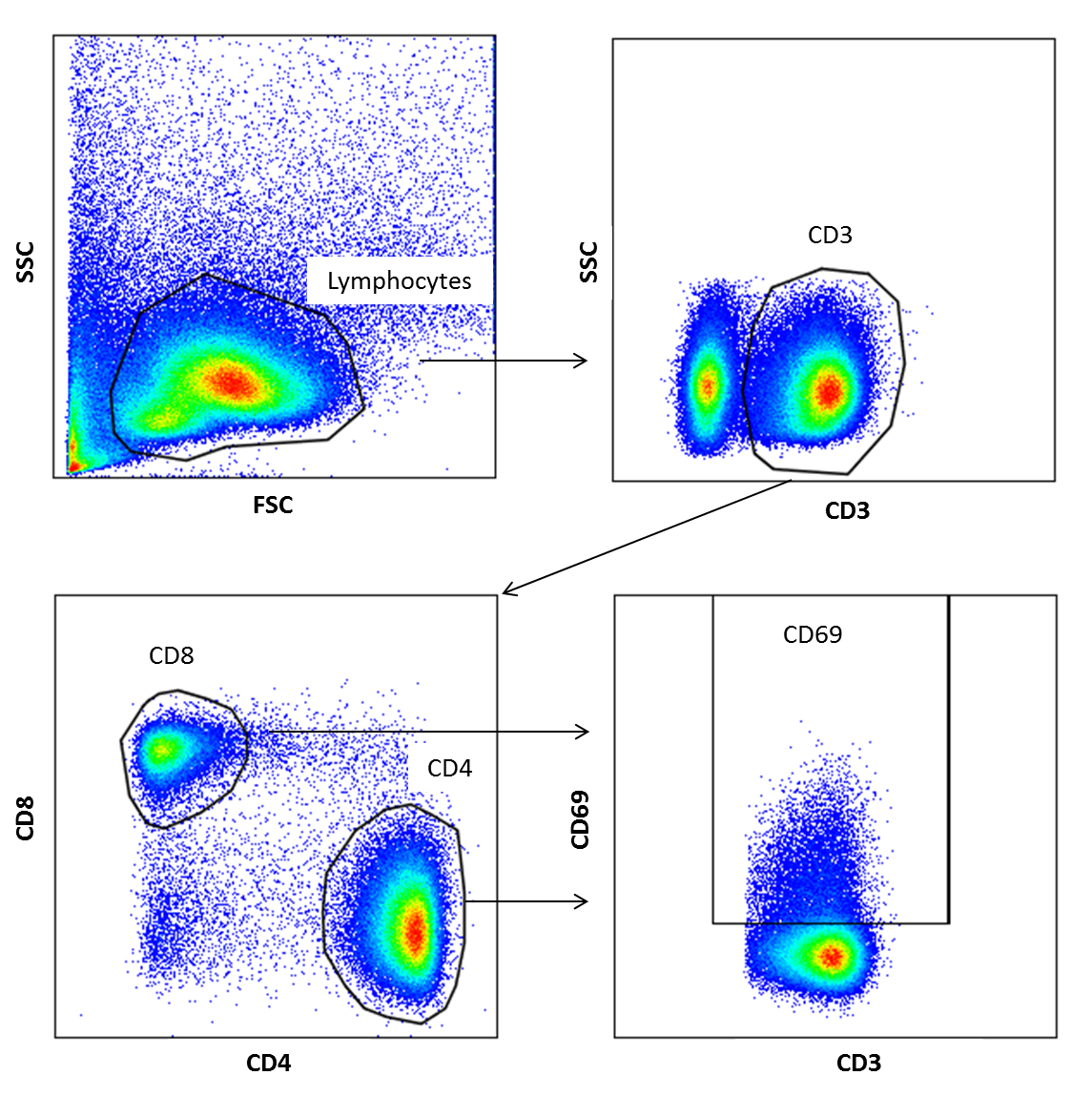


**Supplementary figure S1. Gating strategy for determination of T cell subsets in the spleen.** Lymphocytes were gated based on size and scatter in the forward side scatter plot and T cells were determined by selecting CD3^+^ cells. Within the CD3^+^ cells CD8^+^ and CD4^+^ cells were selected. Within both the CD8^+^ and CD4^+^ population, the percentage of CD69 positive cells were measured. All isotype controls were set at 1%.


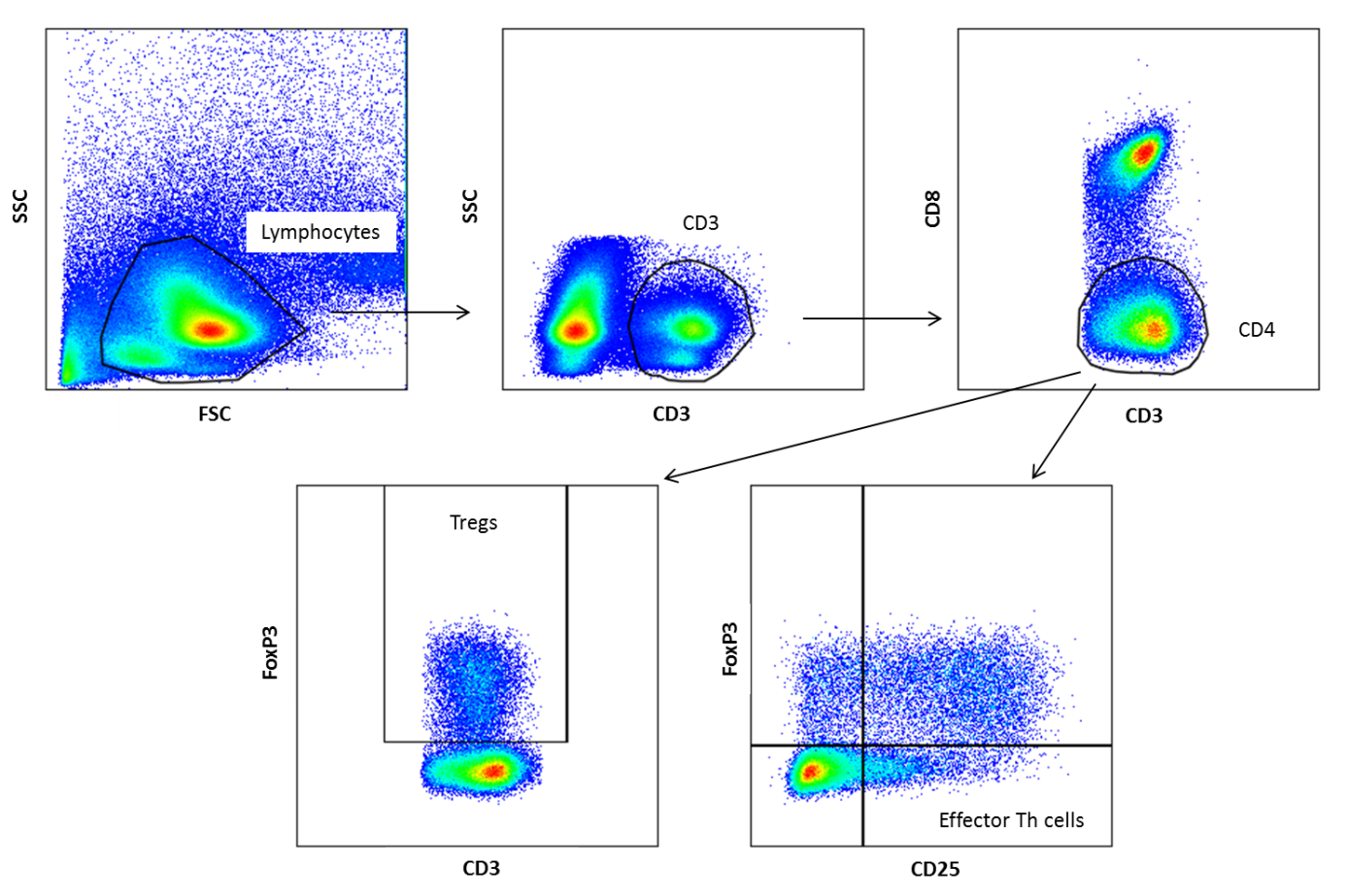


**Supplementary figure S2. Gating strategy for determination of T cell subsets in the spleen.** Lymphocytes were gated based on size and scatter in the forward side scatter plot and T cells were determined by selecting CD3^+^ cells. Within the CD3^+^ cells CD8^+^ and CD8^-^ cells were selected. Within the CD8^-^ population, the percentage of FoxP3^+^ and CD25^+^FoxP3^-^ positive cells were measured. All isotype controls were set at 1%.


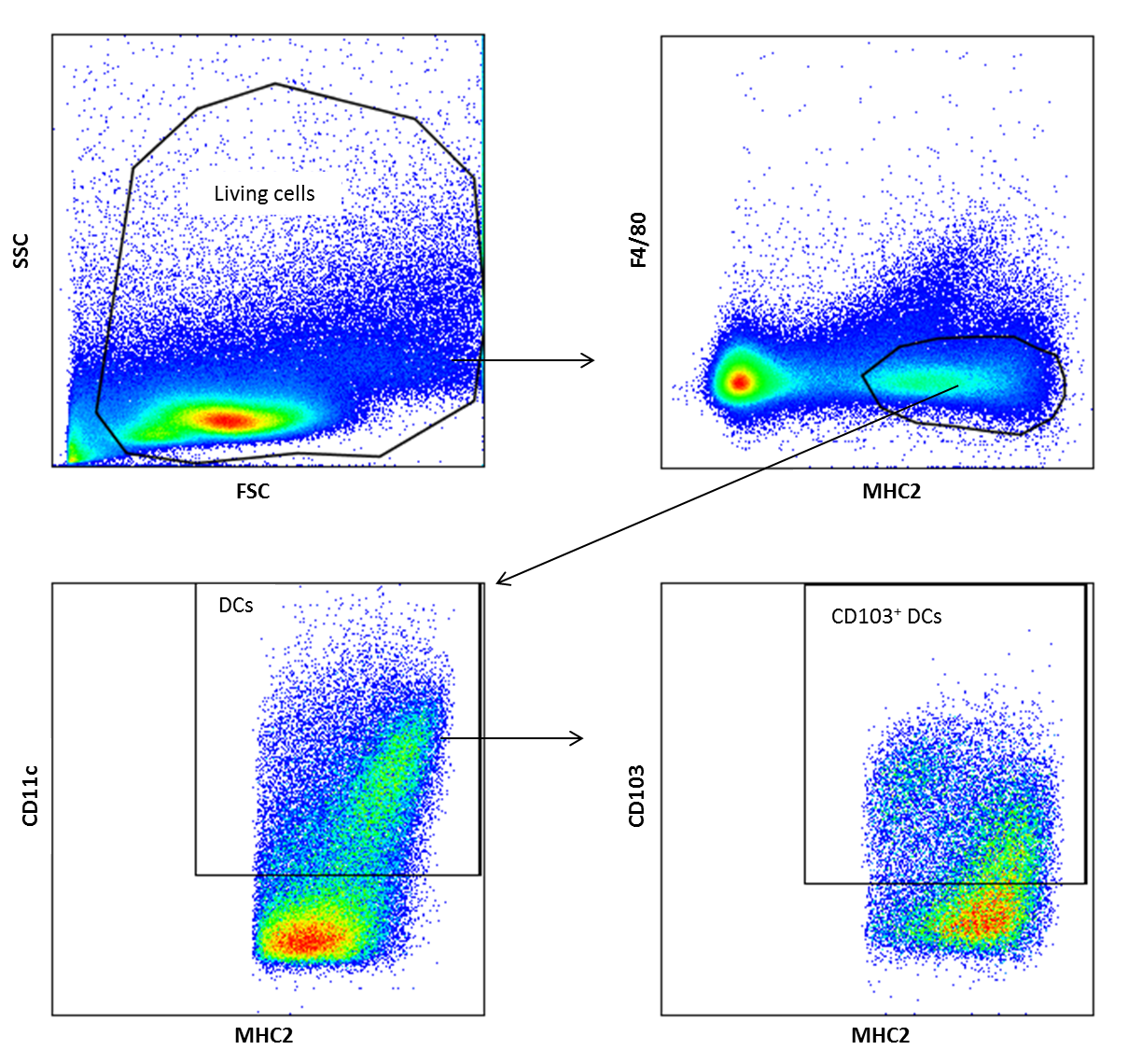


**Supplementary figure S3. Gating strategy for determination of dendritic cells in the mesenteric lymph nodes.** To gate DCs, first living cells were selected based on size in the forward side scatter plot. Within the MHC2^+^ and F4/80^-^, DCs were gated as CD11c^+^. In this population the expression of CD103 was measured. All the isotype controls were set at 1% and these gates were copied to the samples with the antibody mix.

**Supplementary tables**

Supplementary table S1. Selection of immunological diseases and functions that are related to the genes in clusters 2 and 6.

| **Diseases or Functions Annotation** | **Molecules** | **p-Value** |
| --- | --- | --- |
| **Cluster 2** |  |  |
| Quantity of Tc17 cells | IL23R | 3.09E-03 |
| Recognition of natural killer cells | KLRD1 | 3.09E-03 |
| Frequency of Th17 cells | IL23R | 2.14E-02 |
| Accumulation of Th2 cells | RGS16 | 2.45E-02 |
| Activation of gamma-delta T lymphocytes | KLRD1 | 2.45E-02 |
| Migration of Th2 cells | RGS16 | 2.75E-02 |
| Stimulation of Th1 cells | KLRD1 | 3.05E-02 |
| Accumulation of Th17 cells | RGS16 | 3.35E-02 |
| **Cluster 6** |  |  |
| Quantity of phagocytes | GFI1,IFNA4,IRAK3,KIT,PCSK1,PGLYRP1,SOAT1,ST3GAL4,ST6GAL1,TPSG1,TSPAN33 | 4.83E-04 |
| Quantity of antigen presenting cells | IFNA4,KIT,PCSK1,PGLYRP1,SOAT1,ST6GAL1,TPSG1 | 4.33E-03 |
| Production of lymphocytes | DBH,GFI1,KIT | 4.35E-03 |
| Quantity of myeloid cells | GFI1,IRAK3,KIT,PGLYRP1,SOAT1,ST3GAL4,ST6GAL1,TPSG1,TSPAN33,VIPR2 | 4.58E-03 |
| Depletion of leukocytes | IFNA4,KIT,PCSK1 | 5.76E-03 |
| Loss of CD4+ CD8+ T-lymphocytes | PTCH2 | 5.76E-03 |
| Production of Th1 cells | DBH | 5.76E-03 |
| Quantity of neutrophils | GFI1,IRAK3,KIT,PGLYRP1,ST3GAL4,ST6GAL1 | 6.62E-03 |
| Production of T lymphocytes | DBH,KIT | 1.05E-02 |
| Quantity of macrophages | KIT,PGLYRP1,SOAT1,ST6GAL1,TPSG1 | 1.38E-02 |
| Cell tethering or rolling of leukocytes | C1GALT1,CHST4,ST3GAL4 | 1.52E-02 |
| Production of TREG cells | KIT | 2.28E-02 |
| Quantity of inflammatory leukocytes | TPSG1 | 2.28E-02 |
| Function of dendritic cells | GFI1,IRAK3,ST6GAL1 | 2.82E-02 |

Supplementary table S2. Selection of immunological diseases and functions that are related to the genes in clusters 1 and 4.

| **Diseases or Functions Annotation** | **Molecules** | **p-Value** |
| --- | --- | --- |
| **Cluster 1** |  |  |
| Quantity of CD8+ T lymphocyte | CLEC10A,Fcrls,Mt1,Mt2 | 4.66E-03 |
| Chemotaxis by myeloid dendritic cells | Retnlg | 1.56E-02 |
| Quantity of inflammatory leukocytes | F13A1 | 1.56E-02 |
| Regulation of dendritic cells | SOCS2 | 1.56E-02 |
| Suppression of leukocytes | FKBP5,UXT | 2.26E-02 |
| Cell rolling of Th1 cells | GCNT1 | 2.72E-02 |
| Quantity of plasma cells | Mt1,Mt2 | 2.95E-02 |
| Recruitment of phagocytes | CLEC10A,F13A1,GCNT1,TAX1BP1 | 3.75E-02 |
| Differentiation of granulocyte-macrophage progenitor cells | PDGFRA | 3.87E-02 |
| **Cluster 4** |  |  |
| Quantity of peripheral T lymphocyte | BIRC5,BLM | 2.75E-03 |
| Maturation of peripheral T lymphocyte | BIRC5 | 4.92E-03 |
| Quantity of macrocytes | BLM | 4.92E-03 |
| Regulation of inflammatory leukocytes | SPP1 | 4.92E-03 |
| Generation of CD8+ T lymphocyte | SPP1 | 1.47E-02 |
| Number of M1 macrophages | SPP1 | 1.47E-02 |

Supplementary table S3. Selection of immunological diseases and functions that are related to the genes in clusters 3 and 5.

| **Diseases or Functions Annotation** | **Molecules** | **p-Value** |
| --- | --- | --- |
| **Cluster 3** |  |  |
| Activation of lymphocytes | CD38,FLT3,IGHG1,Igkv1-117,IL21R,IRF8,ITK,KLF2,NCR1,NFKBIZ,STAT4,TNFSF13 | 1.84E-07 |
| Cell proliferation of T lymphocytes | BIRC3,CD38,FLT3,HSPA1A/HSPA1B,IGHG1,IL21R,IRF8,ITGAX,ITK,KLF2,STAT4,TNFSF13 | 4.60E-06 |
| Response of lymphatic system cells | CD38,Igkv1-117,IL21R,IRF8,ITK,NCR1,STAT4 | 1.28E-05 |
| Quantity of B lymphocytes | CD38,FLT3,IL21R,IRF8,ITK,KLF2,TGFBI,TNFSF13 | 6.85E-05 |
| Response of lymphocytes | CD38,Igkv1-117,IL21R,ITK,NCR1,STAT4 | 8.06E-05 |
| Quantity of leukocytes | AKT3,BIRC3,CD38,CYP4A11,FLT3,IL21R,IRF8,ITK,KLF2,PDE4B,STAT4,TGFBI,TNFSF13 | 1.03E-04 |
| Response of natural killer cells | CD38,NCR1,STAT4 | 1.04E-04 |
| Quantity of immunoglobulin | IGHG1,IL21R,IRF8,ITK,NFKBIZ,Pvr,TNFSF13 | 1.16E-04 |
| Function of lymphocytes | BIRC3,IL21R,IRF8,ITK,KLF2,STAT4,TNFSF13 | 1.34E-04 |
| Cytotoxicity of T lymphocytes | HSPA1A/HSPA1B,IGHG1,IL21R,STAT4 | 1.39E-04 |
| Development of CD4+ T-lymphocytes | ITK,NFKBIZ | 1.78E-04 |
| Leukocyte migration | CD38,HSPA1A/HSPA1B,IGHG1,IL21R,ITGAX,ITK,KCNE3,KLF2,NCR1,NFKBIZ,PDE4B,TNFSF13 | 3.18E-04 |
| Response of leukemia cell lines | CD38,ITK,KLF2 | 3.39E-04 |
| Function of T lymphocytes | BIRC3,IL21R,IRF8,ITK,KLF2,STAT4 | 3.40E-04 |
| Activation of T lymphocytes | CD38,IGHG1,IL21R,IRF8,ITK,KLF2,STAT4 | 3.91E-04 |
| Differentiation of plasma cells | CD38,IL21R,TNFSF13 | 5.45E-04 |
| Response of helper T lymphocytes | IL21R,ITK,STAT4 | 5.45E-04 |
| Function of leukocytes | BIRC3,IL21R,IRF8,ITK,KLF2,NFKBIZ,STAT4,TNFSF13 | 5.76E-04 |
| Quantity of regulatory T lymphocytes | AKT3,CD38,FLT3,IRF8 | 7.01E-04 |
| Quantity of lymphocytes | AKT3,CD38,FLT3,IL21R,IRF8,ITK,KLF2,STAT4,TGFBI,TNFSF13 | 7.37E-04 |
| Activation of natural killer cells | FLT3,NCR1,NFKBIZ,STAT4 | 1.01E-03 |
| Activation of B lymphocytes | CD38,IGHG1,Igkv1-117,TNFSF13 | 1.11E-03 |
| Activation of naive T lymphocytes | IGHG1,STAT4 | 1.27E-03 |
| Quantity of naive lymphocytes | ITK,TNFSF13 | 1.27E-03 |
| Maturation of leukocytes | HSPA1A/HSPA1B,IL21R,IRF8,ITK,TNFSF13 | 1.35E-03 |
| Differentiation of CD4+ T-lymphocytes | IL21R,ITK,STAT4 | 1.42E-03 |
| Differentiation of B lymphocytes | CD38,FLT3,IL21R,IRF8,TNFSF13 | 1.46E-03 |
| Response of Th2 cells | IL21R,ITK | 2.08E-03 |
| Differentiation of Th2 cells | IGHG1,ITK,STAT4 | 2.55E-03 |
| Function of CD8+ T lymphocyte | IL21R,ITK | 3.08E-03 |
| Quantity of antigen presenting cells | BIRC3,FLT3,IRF8,STAT4,TNFSF13 | 3.80E-03 |
| T cell development | IGHG1,IL21R,IRF8,ITK,KLF2,NFKBIZ,STAT4 | 5.82E-03 |
| Quantity of macrophages | BIRC3,IRF8,STAT4,TNFSF13 | 5.93E-03 |
| Migration of dendritic cells | CD38,IL21R,NCR1 | 6.02E-03 |
| Development of Th17 cells | IL21R,NFKBIZ | 6.22E-03 |
| Quantity of T lymphocytes | AKT3,CD38,FLT3,IL21R,IRF8,ITK,TNFSF13 | 6.30E-03 |
| Differentiation of neutrophils | FLT3,IRF8 | 6.52E-03 |
| Activation of antigen presenting cells | APOH,FLT3,IGHG1,STAT4,TNFSF13 | 7.04E-03 |
| Function of Th1 cells | IL21R,STAT4 | 8.15E-03 |
| Function of Th2 cells | IL21R,STAT4 | 9.58E-03 |
| Development of B lymphocytes | FLT3,IL21R,KLF2 | 1.37E-02 |
| **Cluster 5** |  |  |
| Development of follicular T helper cells | BCL6,MAP3K14 | 1.90E-03 |
| Development of helper T lymphocytes | BCL6,CD226,DLL1,MAP3K14 | 7.48E-04 |
| Development of Th1 cells | BCL6,CD226,DLL1 | 2.40E-04 |
| Differentiation of CD4+ T-lymphocytes | BCL6,CD226,GLI2 | 5.27E-03 |
| Differentiation of follicular T helper cells | BCL6,DLL4 | 3.90E-03 |
| Differentiation of T lymphocytes | BCL6,CD226,DLL1,DLL4,GLI2,GLI3,let-7,SATB1 | 2.77E-03 |
| Differentiation of Th1 cells | BCL6,DLL1,DLL4,GLI2,let-7 | 1.08E-04 |
| Differentiation of Th1-like cells | GLI2 | 4.68E-03 |
| Differentiation of Th2 cells | BCL6,DLL1,DLL4,GLI2,let-7 | 7.25E-05 |
| Frequency of invariant natural killer T cells | ABCG1,CD226 | 6.49E-05 |
| Frequency of lymphocytes | ABCG1,CD226,DLL4 | 7.70E-03 |
| Hyperplasia of regulatory T lymphocytes | MAP3K14 | 9.34E-03 |
| Lymphocyte homeostasis | ABCG1,BCL6,CD226,DLL1,DLL4,GLI2,GLI3,HOXA9,let-7,MAP3K14,PSMB9,SATB1,WT1,ZC3H8 | 2.02E-05 |
| Proliferation of immune cells | ABCG1,BCL6,CD226,DLL4,HOXA9,HR,IDO1,MAP3K14,SATB1,ST8SIA1,WT1 | 1.36E-02 |
| Recognition of dendritic cells | CD226 | 9.34E-03 |
| T cell development | BCL6,CD226,DLL1,DLL4,GLI2,GLI3,HOXA9,let-7,MAP3K14,PSMB9,SATB1,WT1 | 1.68E-04 |
| T cell homeostasis | BCL6,CD226,DLL1,DLL4,GLI2,GLI3,HOXA9,let-7,MAP3K14,PSMB9,SATB1,WT1,ZC3H8 | 4.95E-05 |
| T cell-dependent B cell activation by T lymphocytes | BCL6 | 9.34E-03 |

**Supplementary table S4.** Antibody specifications.

| **Specificity** | **Clone name** | **Fluorchrome** | **Concentration** | **Dilution*^a^*** | **Supplier** |
| --- | --- | --- | --- | --- | --- |
| MHC2 | 2G9 | Biotin  Streptavidin-Pacific Orange | 0.5 mg/ml  1 mg/ml | 200x  100x | ThermoFisher ThermoFisher |
| F4/80 | BM8 | A700 | 0.5 mg/ml | 75x | Biolegend |
| CD11c | N418 | PE-Cy7 | 0.2 mg/ml | 100x | Biolegend |
| CD103 | [2E7](http://www.biolegend.com/index.php?page=pro_sub_cat&action=search_clone&criteria=2E7) | PerCP-Cy5.5 | 0.2 mg/ml | 25x | Biolegend |
| CD3 | 17A2 | Pacific Blue | 0.5 mg/ml | 80x | Biolegend |
| CD8 | 53-6.7 | A700 | 0.5 mg/ml | 50x | Biolegend |
| CD4 | 2E7 | PE-Cy7 | 0.2 mg/ml | 100x | Biolegend |
| CD69 | H1.2F3 | FitC | 0.5 mg/ml | 25x | Biolegend |
| CD25 | PC61 | PE-Cy7 | 0.2 mg/ml | 50x | Biolegend |
| FoxP3 | FJK-16s | FitC | 0.5 mg/ml | 50x | eBioscience |

*^a^* *Dilution used in a total volume of 25 µl. supplemented with PBS + 10% FC*
